# Character strengths as protective factors against depression and suicidality among male and female employees

**DOI:** 10.1186/s12889-018-5997-1

**Published:** 2018-08-31

**Authors:** Hye Ri Kim, Sun Mi Kim, Ji Sun Hong, Doug Hyun Han, Seo-Koo Yoo, Kyung Joon Min, Young Sik Lee

**Affiliations:** 1 Department of Psychiatry, Chung-Ang University Medical Center, 102 Heukseok-ro, Dongjak-gu, Seoul, 06973 South Korea; 2 School of Social Welfare, Soongsil University, 369 Sangdo-ro, Dongjak-gu, Seoul, 06978 South Korea

**Keywords:** Character strengths, Depression, Suicidality, Workplace mental health, Positive psychology

## Abstract

**Background:**

So far, studies on workplace mental health have only focused on work-related environmental risk factors, disregarding both protective and individual factors of employees. Therefore, we aimed to identify character strengths that act as protective factors against depressive moods and suicidality in Korean employees.

**Methods:**

In total, 84 male and 151 female employees (aged 19–50 years) reported their sociodemographic characteristics; depressive symptoms, as measured by the Beck Depression Inventory-II; suicidality, as measured by the Korean version of the MINI International Neuropsychiatric Interview suicidality module; and character strengths, as measured by the 24 Character Strength Alphas on the Values in Action Survey-72. We conducted a hierarchical logistic regression, in which depressive mood and suicidality served as the categorical outcome variables.

**Results:**

In females, scores on the “curiosity” (B = 1.107, Wald = 10.207, odds ratio = 3.026, *p* = .001) and “love” (B = .862, Wald = 5.767, odds ratio = 2.367, *p* = .016) sub scales of the 24 Character Strength Alphas on the Values in Action Survey-72 were statistically significant predictors of having depressive mood. Additionally, females’ scores on “judgment” (B = − 1.405, Wald = 5.663, odds ratio = .245, *p* = .017) and “kindness” (B = − 1.456, Wald = 6.486, odds ratio = .233, *p* = .011) were protective factors against suicidality. In males, the “love” (B = 1.746, Wald = 4.279, odds ratio = 5.729, *p* = .039) score was a predictor of having depressive mood, while “teamwork” (B = − 2.204, Wald = 4.666, odds ratio = .110, *p* = .031) and “creativity” (B = − 1.384, Wald = 4.202, odds ratio = .251, *p* = .040) scores were protective factors against having depressive mood and suicidality, respectively.

**Conclusions:**

We suggest that focusing on “judgement” and “kindness” in female employees, and “teamwork” and “creativity” in male employees, and engaging in activities that use these strengths at the workplace can be protective factors against depression and suicidality. Future research should focus on developing interventions to promote these character strengths among employees at the workplace.

## Background

Since the socioeconomic burden of mental health problems of employees has increased, policymakers and health professionals are demanding a better understanding of the links between work and mental health. So far, various studied have aimed to elucidate the complex relationship between work environment and mental health. According to a meta-analysis, the development of depression among employees is significantly related to high job strain, low job control, and bullying [1]. Another meta-analysis reported that high work demands, low autonomy, lack of reward, and job insecurity increase the risk of burnout [2] . In a study conducted in South Korea, lack of social support, job insecurity, and uncomfortable workplace environment were found to be risk factors for depression [3, 4]. In a systematic review study, the following three categories of work-related factors that contribute to the development of mental health problems were identified: unbalanced employment design, job uncertainty, devaluation, and disrespect in the workplace [5].

However, until now, studies on workplace mental health have focused on work-related environmental risk factors, disregarding both protective and individual factors of employees. The identification and promotion of protective factors is crucial because they reduce the risk for mental problems. A few existing studies have focused on protective factors. It has been shown that high levels of job support, workplace justice, and job control are protective factors against burnout symptoms, and that job control is also protective against depression [1, 2]. In addition, not only work-related environmental factors, but also the individual factors of employees, including socioeconomic status, marriage and family environment, years of education, temperament, personality, and strengths, should be considered while assessing the mental health of employees at work.

Positive psychology focuses on concepts such as character strengths and virtues [6]. It aims to promote positive emotions through utilizing these positive traits [7]. Virtues are the essential properties of a good character, while character strengths are more specific and measurable examples of virtues that are manifested in the thoughts, feelings, and behaviours of individuals [6]. Twenty-four character strengths are assigned to six superior-level virtues, as follows: (1) *“wisdom and knowledge,”* which includes the character strengths of “creativity,” “curiosity,” “judgment,” “love of learning,” and “perspective”; (2) *“courage,”* which includes the character strengths of “bravery,” “perseverance,” “honesty,” and “zest”; (3) *“humanity,”* which includes the character strengths of “love,” “kindness,” and “social intelligence”; (4) *“justice,”* which includes the character strengths of “teamwork,” “fairness,” and “leadership”; (5) *“temperance,”* which includes the character strengths of “forgiveness,” “humility,” “prudence,” and “self-regulation”; and (6) *“transcendence,”* a virtue that includes the character strengths of “appreciation of beauty and excellence,” “gratitude,” “hope,” “humour,” and “spirituality.”

Previous studies have reported gender differences in the prevalence of psychiatric disorders [8, 9]. According to a large-scale study conducted in 10 European countries, the prevalence of internalizing disorders, such as depression and anxiety disorders, was higher in women than in men, and the prevalence of externalizing disorders, such as substance addiction and conduct disorder, was higher in men than in women [9]. It has also been reported that suicidal ideations, plans, and attempts are more common in women than in men [9]. Biological and psychosocial factors work together to cause these differences [10]. A study on gender differences in the causes of depression showed that depression was associated with physical health, unemployment, and workplace problems in men, while interpersonal problems, family illness and death, and pregnancy contributed to the development of depression in women [8]. In a study of 53,969 Korean workers, low job control and lack of reward were positively associated with suicidal thoughts in men, and interpersonal problems were associated with higher risk of suicidal thoughts in women [11]. Further, in previous studies on the gender gap in character strengths, men showed high scores on “bravery” and “creativity,” while women showed high scores on “love” and “kindness” [12, 13].

Based on these findings, the present study aimed to identify character strengths that act as protective factors against depressive mood and suicidality in male and female employees. Given that high levels of job support, workplace justice, and job control were identified as protective factors against burnout and depression among workers in a previous study [1, 2], we hypothesized that character strengths identified as aspects of the virtues *“humanity”, “justice”,* and/or *“wisdom and knowledge”* are protective factors against depression in employees. In addition, given that low job control and lack of reward were positively associated to the suicidal thoughts in men [11], we hypothesized that character strengths identified as aspects of the virtue *“wisdom and knowledge”* are protective factors against suicidality in male employees. Finally, given that interpersonal problems were associated with higher risk of suicidal thoughts in women in a previous study [11], we hypothesized that character strengths identified as aspects of the virtues *“humanity”* and *“justice”* are protective factors against suicidality in female employees.

## Methods

### Participants and data collection

Participants were employees aged 19 to 50 years, working in the department of customer service of two private organizations in Seoul. We distributed a self-report questionnaire to 270 (male/female: 98/172) employees from March to August 2017. Responses were collected from 251 (male/female: 91/160) employees, with 235 valid responses (male/female: 84/151, active response rate: 93.6%).

This survey was conducted as part of the annual workplace mental health screening project conducted in cooperation with a regional public mental health welfare centre. Therefore, participants were contacted, and the self-reported questionnaire was distributed and collected by the personnel in public mental health centres and in companies, under the planning and supervision of psychiatrists from the Chung-Ang University Hospital.

To minimize social desirability bias, participants were informed that all mental health data will be kept confidential by company officials, and that the results would be sealed and delivered only to the participants themselves. In addition, all participants were informed in advance that they could be used as research data in anonymity-protected situations. The results of the mental health screening were provided as a sealed document to each employee. The document included information on the mental health status of the employee, recommended whether counselling or treatment was required, and explained the treatment access path.

This survey study was approved by the Chung-Ang University Institutional Review Board. Written informed consent was obtained from all individual participants included in the study.

### Variables collected

Demographic information was collected using independent variables including age, sex, years of education, family income (with scores of 1 to 7 for income levels ranging from below $1000 to above $6000 per month), and job grade (low: staff, high: assistant manager/manager/deputy general manager/general manager).

The Beck Depression Inventory (BDI-II) is a 21-item self-reported questionnaire that assesses the severity of depressive symptoms [14]. The total BDI-II score ranges from 0 to 63 points. In this study, “having depressive mood” was defined as having a BDI-II score ≥ 13 points, which represented mild to severe depressive symptoms [14]. The Korean version of the MINI International Neuropsychiatric Interview (MINI) suicidality module (K-MINI) is composed of six questions regarding suicidal ideation and suicide attempts, with different weighed scores ranging from 1 to 10 [15, 16]. The six questions of K-MINI include “Item 1: wish to be dead (1 point),” “Item 2: want to hurt oneself (2 points),” “Item 3: suicidal ideation (6 points),” “Item 4: suicidal plan (10 points),” “Item 5: suicide attempt (10 points),” and “Item 6: suicide attempt ever (4 points).” Item 1–5 are responded to with reference to whether they were experienced during the past one month. The total K-MINI score ranges from 0 to 33. In a previous study, among acute psychiatric patients, the cut-off point for self-harm has been suggested to be six [17] and that for suicide attempts in acute psychiatric emergency departments was suggested to be ten [18]. In the present study, “having suicidality” was defined as having a K-MINI score ≥ 4 points, which represents greater severity than passive suicidal ideation (Item 1) and non-suicidal self-injury (Item 2). This criterion was used because our study participants comprised general working individuals, not psychiatric patients, and because previous suicide attempt is one of the most important factors to predict the high-risk characteristics of suicidality [19]. Previous studies have established the high internal consistency and validity of the BDI-II and K-MINI [14–16].

We measured character strengths and virtues using the 24 Character Strength Alphas Values in Action (VIA) Survey-72 [6]. In this study, all participants completed the VIA-72, and the scores on each subscale of the 24 character strengths were used for the analysis. The VIA-72 was derived from the original VIA Inventory of Strengths (VIA-IS) [6], by extracting the three most internally consistent items from each subscale. The VIA-IS has acceptable internal consistency and test–retest reliability [6, 20]. All scales have satisfactory Cronbach’s alphas (>.70), and the test–retest correlations for all scales over a four-month period are substantial (>.70) [6, 20]. The VIA Institute considers the VIA-72 substantially equivalent to the original long version of the VIA-IS considering the internal consistency reliability and validity measures described [6]. The VIA-72 is a 72-item self-report questionnaire on which respondents report the extent to which statements apply to them. Items are rated on a five-point Likert scale ranging from 1 (very much unlike me) to 5 (very much like me). The subscale score for each of the 24 strengths is calculated by dividing the score by three (the number of questions). Thus, each subscale score has a potential range of 1 through 5, with higher scores indicating higher degree of endorsement of a specific strength. In the present study, the following 11 character strengths from the three virtues were included in the statistical analyses: “creativity,” “curiosity,” “judgment,” “love of learning,” and “perspective” from the virtue *“wisdom and knowledge”*; “love,” “kindness,” and “social intelligence” from the virtue *“humanity”*; and “teamwork,” “fairness,” and “leadership” from the virtue *“justice.”*

### Statistical analysis

The distribution of sociodemographic and clinical characteristics of the study participants was presented using counts and frequencies. Gender differences in characteristics were analysed using the Chi-square and independent t-test. Additionally, we conducted hierarchical logistic regression analyses in which “having depressive mood” and “having suicidality” served as categorical outcome variables, respectively. In the two regression models, age, years of education, family income, and job grade were entered first (Step 1), followed by character strengths (Step 2). We had considered using the BDI-II and K-MINI scores as metric dependent variables in hierarchical linear regression analyses. However, we decided to use “having depressive mood” and “having suicidality” served as categorical outcome variables instead because the total scores on both the BDI-II and K-MINI were not normally distributed. These hierarchical logistic regression analyses were repeated for both genders. For all analyses, the level of significance was set at .05. We conducted all analyses using the Complex Samples module of the PASW Statistics package, version 19 (SPSS Inc., Chicago, IL, USA).

## Results

### Sociodemographic and clinical characteristics of the study population

Table 1 summarizes the descriptive information on the sociodemographic factors, level of depressive symptoms, and level of suicidality. The mean age of the sample was 32.5 ± 6.5 years, and 96.5% of the study population was in the low job grade (staff). The mean BDI-II score was 13.3 ± 10.0, and the mean K-MINI score was 1.5 ± 3.2. Further, 43% of the participants were identified as having depressive mood (BDI-II score ≥ 13) and 21.3% of them were identified as having suicidality (K-MINI score ≥ 4). Female employees reported higher family income (*p* = .029) and BDI-II scores (*p* = .001) than did male employees. More female employees reported having depressive mood (BDI-II score ≥ 13) than did male employees (*p* = .002). Female employees reported higher scores on “creativity” (*p* = .004), “judgment” (*p* = 0.010), and “social intelligence” (*p* = .042) than did male employees.

**Table 1:** Sociodemographic and clinical characteristics of participants (*N* = 235; 84 males and 151 females)

Variables	Total		Female	Male	Statistics
	Mean (SD), N (%)	Range	Mean (SD), N (%)	Mean (SD), N (%)	t/x^2^, *p*
Age, year	32.5 (6.5)	19–50	32.5 (6.8)	32.4 (5.9)	.120, .905
Years of education	10.8 (5.7)	9–18	11.0 (5.6)	10.6 (5.8)	.458, .647
Family income (1 to 7)^1^	4.9 (1.5)	1–7	5.0 (1.5)	4.6 (1.4)	2.196, .029^*^
Job grade (low)^2^	227 (96.5%)		147 (93.4%)	80 (95.2%)	.733, .392
BDI-II score	13.3 (10.0)	0–43	14.9 (10.3)	10.5 (8.9)	3.339, .001^**^
“Having depressive mood” group^3^	101 (43.0%)		76 (50.3%)	25 (29.8%)	9.318, .002^**^
K-MINI score	1.5 (3.2)	0–23	1.6 (3.3)	1.2 (3.0)	.933, .352
“Having suicidality” group^4^	50 (21.3%)		35 (23.2%)	15 (17.9%)	.913, .339
VIA-72 scores					
Creativity	3.0 (.9)	1.0–5.0	3.1 (.9)	2.8 (.8)	2.912, .004^**^
Curiosity	3.0 (.9)	1.0–5.0	3.0 (.9)	2.9 (.8)	1.620, .107
Judgment	2.6 (.7)	1.0–4.7	2.7 (.6)	2.5 (.7)	2.601, .010^*^
Love of learning	3.5 (.9)	1.0–5.0	3.5 (.9)	3.4 (.9)	.733, .464
Perspective	2.8 (.7)	1.0–5.0	2.8 (.8)	2.7 (.7)	.719, .473
Love	2.3 (.8)	1.0–5.0	2.4 (.8)	2.3 (.8)	.706, .481
Kindness	2.4 (.6)	1.0–4.7	2.4 (.6)	2.4 (.6)	.869, .386
Social intelligence	2.6 (.6)	1.0–4.7	2.6 (.6)	2.4 (.6)	2.046, .042^*^
Teamwork	2.4 (.6)	1.0–4.0	2.5 (.6)	2.4 (.6)	1.056, .292
Fairness	2.3 (.6)	1.0–4.0	2.3 (.6)	2.3 (.7)	.123, .903
Leadership	2.6 (.7)	1.0–4.3	2.6 (.7)	2.5 (.6)	1.494, .137

^*^p < .05, ^**^p < .01, ^***^p < .001. BDI-II, Beck Depression Inventory-II; K-MINI, Korean version of the MINI International Neuropsychiatric Interview suicidality module; VIA-72, 24 Character Strength Alphas Values in Action Survey-72

^1^1 to 7: below $1000 to above $6000 per month

^2^Low: staff, high: assistant manager/manager/deputy general manager

^3^“Having depressive mood” group: BDI-II ≥13

^4^“Having suicidality” group: K-MINI ≥4

### Results of the hierarchical logistic regression analysis with having depressive mood as a dependent variable

Results of the hierarchical logistic regression analyses that tested the association of having depressive mood with socio-demographic factors and character strengths measured by the VIA-72 for female and male employees have been summarized in Tables 2 and 3, respectively.

**Table 2:** Results of the hierarchical logistic regression analyses with having depressive mood as a dependent variable, for female employees (*N* = 151)

Independent variables	Model 1				Model 2			OR
	B	Wald	Sig.	OR	B	Wald	Sig.	
Socio-demographic factors								
Age	−.071	7.082	.008^**^	.931	−.082	6.870	.009^**^	.922
Years of education	.033	1.085	.298	1.033	.023	.420	.517	1.023
Family income	−.105	.812	.368	.900	.007	.003	.958	1.007
Job grade (low)	−1.083	.853	.356	.338	−.216	.029	.865	.805
Character strengths measured by the VIA-72								
Creativity					−.651	3.324	.068	.521
Curiosity					1.107	10.207	.001^**^	3.026
Judgment					−.041	.008	.929	.960
Love of learning					−.019	.004	.948	.981
Perspective					−.111	.072	.789	.895
Love					.862	5.767	.016^*^	2.367
Kindness					−.596	1.635	.201	.551
Social intelligence					−.003	.000	.994	.997
Teamwork					−.321	.602	.438	.726
Fairness					−.489	1.035	.309	.613
Leadership					.068	.024	.878	1.071
Statistics of the model								
-2LL	191.244				166.292			
Model χ^2^	9.762 (df = 4), *p* = .045^*^				34.714 (df = 15), *p* = .003^**^			
Step χ^2^	9.762 (df = 4), *p* = .045^*^				24.951 (df = 11), *p* = .009^**^			
Nagelkerke R^2^	.087				.284			
Classification Accuracy (%)	62.1				69.0			

^*^p < .05, ^**^p < .01, ^***^p < .001. VIA-72, 24 Character Strength Alphas Values in Action Survey-72; Sig., significance; OR, odds ratio

**Table 3:** Results of the hierarchical logistic regression analyses with having depressive mood as a dependent variable, for male employees (*N* = 84)

Independent variables	Model 1				Model 2			
	B	Wald	Sig.	OR	B	Wald	Sig.	OR
Socio-demographic factors								
Age	−.015	.119	.731	.986	−.043	.647	.421	.958
Years of education	.020	.211	.646	1.020	.000	.000	.994	1.000
Family income	−.124	.459	.498	.884	−.039	.026	.873	.961
Job grade (low)	−20.339	.000	.999	.000	−20.843	.000	.999	.000
Character strengths measured by the VIA-72								
Creativity					−.816	2.917	.088	.442
Curiosity					.927	1.987	.159	2.527
Judgment					.186	.098	.754	1.204
Love of learning					−.479	1.746	.186	.619
Perspective					−.159	.051	.822	.853
Love					1.746	4.279	.039^*^	5.729
Kindness					.248	.127	.721	1.282
Social intelligence					−.176	.075	.785	.838
Teamwork					−2.204	4.666	.031^*^	.110
Fairness					.684	.958	.328	1.982
Leadership					−.398	.400	.527	.672
Statistics of the model								
-2LL	97.972				81.454			
Model χ^2^	3.600 (df = 4), *p* = .463				20.118 (df = 15), *p* = .167			
Step χ^2^	3.600 (df = 4), *p* = .463				16.518 (df = 11), *p* = .123			
Nagelkerke R^2^	.060				.305			
Classification Accuracy (%)	69.9				71.1			

^*^p < .05, ^**^p < .01, ^***^p < .001. VIA-72, 24 Character Strength Alphas Values in Action Survey-72; Sig., significance; OR, odds ratio

### Statistical results for female employees

For female employees, both Model 1 and 2 of the hierarchical logistic regression analysis revealed a significant overall model fit. In Analysis Model 2, both model χ^2^ (34.714, *p* = .003) and the Nagelkerke R^2^ value (.284, explaining about 28.4% of the variance in the dependent variable “having depressive mood”) indicated that the model was good enough to predict having depressive mood. When the practical usefulness of the model was examined based on the classification accuracy, 15 variables in Analysis Model 2 considerably enhanced its prediction accuracy up to 69.0% for the group membership of the dependent variable. Wald statistics were used to confirm if each indicator had a significant individual relationship with having depressive mood. Among the independent variables, the character strengths “curiosity” (B = 1.107, Wald = 10.207, odds ratio = 3.026, *p* = .001) and “love” (B = .862, Wald = 5.767, odds ratio = 2.367, *p* = .016) were statistically significant predictors of having depressive mood. Otherwise, greater age (B = −.082, Wald = 6.870, odds ratio = .922, *p* = .009) was a statistically significant protective factor for having depressive mood. After accounting for the influence of other variables, the odds ratio of 3.026 for “curiosity” indicates that an increase of one unit in the “curiosity” score increases the probability of having depressive mood by 3.026 times. Additionally, the odds ratio of 2.367 for “love” means that an increase of one unit in the “love” score increases the probability of having depressive mood by 2.367 times.

### Statistical results for male employees

For male employees, both Model 1 and 2 of the hierarchical logistic regression analysis revealed statistically nonsignificant overall model fit. However, in Analysis Model 2, the Nagelkerke R^2^ value was .305, explaining about 30.5% of the variance in the dependent variable “having depressive mood.” In addition, when the practical usefulness of the model was examined based on the classification accuracy, 15 variables in Analysis Model 2 considerably enhanced its prediction accuracy up to 71.1% for the group membership of the dependent variable. Among the independent variables, the character strength “love” was a statistically significant predictor of having depressive mood (B = 1.746, Wald = 4.279, odds ratio = 5.729, *p* = .039). Otherwise, the character strength “teamwork” was a statistically significant protective factor for having depressive mood (B = − 2.204, Wald = 4.666, odds ratio = .110, *p* = .031). After accounting for the influence of other variables, the odds ratio of 5.729 for “love” means that an increase of one unit in the “love” score increases the probability of having depressive mood by 5.729 times. Otherwise, the odds ratio of .110 for “teamwork” means that an increase of one unit on the “teamwork” score decreases the probability of having depressive mood by 9.091 (1/.110) times.

### Results of the hierarchical logistic regression analysis with having suicidality as the dependent variable

Results of the hierarchical logistic regression analyses that tested the association of having suicidality with socio-demographic factors and character strengths measured by the VIA-72 for female and male employees have been summarized in Tables 4 and 5, respectively.

**Table 4:** Results of the hierarchical logistic regression analyses with having suicidality as a dependent variable, for female employees (*N* = 151)

Independent variables	Model 1				Model 2			
	B	Wald	Sig.	OR	B	Wald	Sig.	OR
Socio-demographic factors								
Age	−.045	2.276	.131	.956	−.063	2.779	.095	.939
Years of education	.015	.173	.677	1.016	.014	.107	.744	1.014
Family income	−.142	1.090	.296	.867	−.268	2.643	.104	.765
Job grade (low)	.098	.007	.934	1.103	−.107	.006	.936	.899
Character strengths measured by the VIA-72								
Creativity					.772	3.414	.065	2.165
Curiosity					.068	.037	.848	1.070
Judgment					−1.405	5.663	.017^*^	.245
Love of learning					−.072	.043	.836	.930
Perspective					.056	.015	.901	1.058
Love					.757	3.778	.052^†^	2.133
Kindness					−1.456	6.486	.011^*^	.233
Social intelligence					−.148	.089	.765	.863
Teamwork					.005	.000	.991	1.005
Fairness					.663	1.372	.242	1.940
Leadership					.518	.960	.327	1.679
Statistics of the model								
-2LL	154.947				127.425			
Model χ^2^	2.998 (df = 4), *p* = .558				30.520 (df = 15), *p* = .010^*^			
Step χ^2^	2.998 (df = 4), *p* = .558				27.522 (df = 11), *p* = .004^**^			
Nagelkerke R^2^	.031				.286			
Classification Accuracy (%)	76.6				80.7			

^*^p < .05, ^**^p < .01, ^***^p < .001, ^†^borderline significance. VIA-72, 24 Character Strength Alphas Values in Action Survey-72; Sig., significance; OR, odds ratio

**Table 5:** Results of the hierarchical logistic regression analyses with having suicidality as a dependent variable, for male employees (*N* = 84)

Independent variables	Model 1				Model 2			
	B	Wald	Sig.	OR	B	Wald	Sig.	OR
Socio-demographic factors								
Age	−.040	.638	.424	.961	−.101	2.004	.157	.904
Years of education	.028	.289	.591	1.028	−.033	.241	.623	.967
Family income	−.366	2.434	.119	.694	−.131	.154	.695	.877
Job grade (low)	−19.371	.000	.999	.000	−19.238	.000	.999	.000
Character strengths measured by the VIA-72								
Creativity					−1.384	4.202	.040^*^	.251
Curiosity					1.709	3.389	.066	5.525
Judgment					.754	.903	.342	2.125
Love of learning					−.520	1.045	.307	.595
Perspective					1.181	1.308	.253	3.259
Love					1.162	.965	.326	3.198
Kindness					.336	.122	.726	1.400
Social intelligence					−.626	.443	.506	.535
Teamwork					−1.967	2.417	.120	.140
Fairness					.028	.001	.974	1.028
Leadership					− 1.057	1.278	.258	.348
Statistics of the model								
-2LL	73.690				52.452			
Model χ^2^	4.743 (df = 4), *p* = .315				25.981 (df = 15), *p* = .038^*^			
Step χ^2^	4.743 (df = 4), *p* = .315				21.238 (df = 11), *p* = .031^*^			
Nagelkerke R^2^	.091				.440			
Classification Accuracy (%)	81.9				90.4			

^*^p < .05, ^**^p < .01, ^***^p < .001. VIA-72, 24 Character Strength Alphas Values in Action Survey-72; Sig., significance; OR, odds ratio

### Statistical results for female employees

For female employees, Model 2 of the hierarchical logistic regression analyses revealed a significant overall model fit. In Analysis Model 2, both model χ^2^ (30.520, *p* = .010) and the Nagelkerke R^2^ value (.286, explaining about 28.6% of the variance in the dependent variable “having suicidality”) indicated that the analysis model was good enough to predict having suicidality. When the practical usefulness of the model was examined based on the classification accuracy, 15 variables in Analysis Model 2 considerably enhanced its prediction accuracy up to 80.7% for the group membership of the dependent variable. Wald statistics were used to determine if each indicator had a significant individual relationship with having suicidality. Among the independent variables, the character strengths “love” was a predictor of having suicidality with a marginal significance (B = .757, Wald = 3.778, odds ratio = 2.133, *p* = .052). Otherwise, the character strengths “judgment” (B = − 1.405, Wald = 5.663, odds ratio = .245, *p* = .017) and “kindness” (B = − 1.456, Wald = 6.486, odds ratio = .233, *p* = .011) were statistically significant protective factors for having suicidality. After accounting for the influence of other variables, the odds ratio of 2.133 for “love” means that an increase of one unit in the “love” score increases the probability of having suicidality by 2.133 times. Otherwise, the odds ratio of .245 for “judgement” means that an increase of one unit in the “judgement” score decreases the probability of having suicidality by 4.082 (1/.245) times. In addition, the odds ratio of .233 for “kindness” means that an increase of one unit in the “kindness” score decreases the probability of having suicidality by 4.292 (1/.233) times.

### Statistical results for male employees

For male employees, Model 2 of the hierarchical logistic regression analyses revealed a significant overall model fit. In Analysis Model 2, both model χ^2^ (25.981, *p* = .038) and the Nagelkerke R^2^ value (.440, explaining about 44.0% of the variance in the dependent variable “having suicidality”) indicated that the analysis model was good enough to predict having suicidality. When the practical usefulness of the model was examined based on the classification accuracy, 15 variables in Analysis Model 2 considerably enhanced its prediction accuracy up to 90.4% for the group membership of the dependent variable. Among the independent variables, the character strength “creativity” was a statistically significant protective factor for having suicidality (B = − 1.384, Wald = 4.202, odds ratio = .251, *p* = .040). After accounting for the influence of other variables, the odds ratio of .251 for “creativity” means that an increase of one unit in the “creativity” score decreases the probability of having suicidality by 3.98 (1/.251) times.

## Discussion

The present study aimed to identify the character strengths that act as protective factors against depressive mood and suicidality in Korean employees. In summary, the character strengths “judgment” and “kindness” were identified as protective factors against suicidality in female employees, while “teamwork” and “creativity” were found to be protective factors against depressive mood and suicidality, respectively, in male employees. On the other hand, the character strengths “curiosity” and “love” were identified as risk factors for depressive mood in female employees, while “love” played the same role in male employees. To our knowledge, this is the first study to investigate the associations of character strengths with depressive mood and suicidality in employees.

In this study, female employees reported a higher level of depression as compared to male employees, but there was no gender difference in the incidence of suicidality. So far, no clear explanation has been found for gender differences in the level of stress and depression experienced in the workplace. One explanation could be that women may experience conflicts between work and family more than men would, with small but significant gender differences. Higher female stress levels seem to be related to the multiple roles they are expected to fulfil [21, 22]. In the present study, female employees reported higher scores on “creativity”, “judgment”, and “social intelligence” than did male employees. Similar to the present findings, a previous study on gender differences in character strengths showed that women typically reported higher scores on strength indicators than did men [13]. However, the detailed results on gender differences differed from a previous study conducted on a large general population sample from the UK [13], which reported that scores on “kindness,” “love,” and “gratitude” were higher among women. Additionally, this study reported a large effect size for gender differences. The differences in the findings of this study and the present study may be attributed to the fact that the present sample comprised employees with a younger mean age as compared to the previous study.

According to the present results, “judgement” and “kindness” are character strengths that function as protective factors against suicidality in females. “Judgement” is included in the virtue *“wisdom and knowledge”,* which represents intellectual strength. Intellectual strength plays an important role in choosing adaptive coping strategies and avoiding maladaptive coping strategies in the workplace [23]. An adaptive coping strategy can help individuals to spend their energies on addressing the cause of stress effectively or accomplishing their intended goal [24]. Intellectual strength can provide psychological resources for individuals to use and cope when faced with stress. These strengths provide self-efficacy and a sense of control as they perform new tasks and, thus, reduce stress [25, 26]. “Kindness” is included in the virtue *“humanity”,* and it seems to be an important strength for the creation of a comfortable work atmosphere and for building good relationships with colleagues as well as customers. Previous research has shown that interpersonal strengths, including “kindness,” “teamwork,” and “humour” are associated with reduced negative coping in various types of workers [23]. In a previous systematic review and meta-analysis on the effect of workplace environment on burnout, along with high levels of workplace justice and job control, high levels of social support from co-workers was found to have a protective effect against burnout syndrome [2]. In addition, in a study on radiologists, social support in the workplace was reported as a protective factor against depression and anxiety [27]. The participants in the present study were employees from the customer service department of private organizations. Therefore, employees’ “kindness” was protective against burnout from customers’ complaints and conflict with customers, and it may promote their emotional and operational fulfilment.

According to the present results, the character strengths “teamwork” and “creativity” are protective factors against depressive mood and suicidality, respectively. “Teamwork” is also known as citizenship, and it is included in the virtue *“justice”,* along with “fairness” and “leadership”. Workplace research shows that “teamwork” is the most important predictor of work-related performance and overall workplace well-being [28, 29]. “Creativity” is included in the virtue *“wisdom and knowledge”,* which represents intellectual strength, and “judgement” were found to function as protective factors against suicidality in females. For male employees, social support in the form of good relationships with colleagues, upholding justice based on citizenship (“teamwork”), and the self-efficacy and sense of control based on abundant psychological resources (“creativity”) might be helpful in reducing depressive mood and suicidality.

On the other hand, in the present study, the character strength “love” was identified as a risk factor for depressive mood in female employees. “Love” is included in the virtue *“humanity”,* which refers to the social strength required to care for others and make friends. It is unclear why “love” was identified as a risk factor for depressive mood in female employees. We cautiously suggest that this character strength seems to commonly describe an individual’s character as emotional rather than rational, or as sensitive to rewards from the external world. Because positive psychology focuses on improving the happiness of healthy people rather than that of psychiatric patients, little research has been conducted on the associations among character strengths, depression, and suicidality. Instead, we refer to studies that used the Temperament and Character Inventory (TCI) to investigate the associations between personality and depression (as well as suicidality) [30–33]. The present results are consistent with the findings of previous TCI studies in which healthy control groups were characterized by a greater capability for self-regulation through cognitive control than were depressed groups, and depressive groups were found to be more likely to have a temperament related to emotional instability [30–33].

Ironically, in the present study, “curiosity,” which is included in the virtue *“wisdom and knowledge”* along with “creativity” and “judgment,” was identified as a risk factor for depression in female workers, although curiosity has been reported as an important strength associated with vitality and adaptation in previous studies [34]. This may be because most of the participants included in the present study belonged to a low job grade. Therefore, they may have lesser opportunities to use the strengths of “curiosity” as a leader. Rather, they may often be expected to act as a dedicated teammate and subordinate who follows orders. Therefore, the present findings need to be verified through future replication studies.

Because character strengths are commensurate with individuals’ traits, an individual already possesses a given strength to a certain degree [35]. Therefore, it might be possible to refer to character strengths as causal factors—whether protective or risk factors—for depressive mood and suicidality at some level. However, the expression of traits is changeable depending on an individual’s motivation to behave in a certain way in a certain environment [36]. Therefore, the identification of character strengths as protective factors against depressive mood and suicidality in employees would be helpful for creating a better understanding of these mental conditions. Positive psychology in the workplace focuses on building strengths rather than on correcting weaknesses [37]. Positive institutions are those that focus on developing individual strengths [7]. The application of an individual’s strengths to a job is known to improve life and job satisfaction, well-being, and the individual’s personal meaning of life [38]. According to previous meta-analysis and review articles, strength-based interventions not only promote positive emotions but also reduce depressive mood and suicidality [39, 40]. Interventions that use and aim to strengthen the signature strengths of “hope” and “gratitude” every day have been effective in decreasing depression [35, 41, 42]. Positive psychology interventions related to “gratitude,” such as writing gratitude letters and actively counting blessings, and interventions using individuals’ character strengths have been shown to be feasible and effective for inpatients with suicidal ideation [43]. Still, few positive psychology-based interventions have been applied to the workplace. However, the findings herein suggest that positive psychology-based interventions could be effective in organizations as well.

Making an effort to focus on the character strengths of employees and to engage in activities that use employee strengths would be effective for the promotion of job satisfaction and workplace well-being. A workplace intervention trial revealed that the use of highest strengths at work significantly increases job performance and harmonious passion [44]. In a controlled study, strength intervention at the workplace was found to promote positive mood in comparison with waiting list controls [45]. Based on the results of the present is study, we suggest that focusing on “judgement” and “kindness” in female employees, and on “teamwork” and “creativity” in male employees, as well as engaging in activities that use these strengths at the workplace can be protective against depression and suicidality. For example, following the work of organizations can promote employee strengths at work by ensuring individual–organizational fit in recruitment and job-sharing processes, training and educating employees, encouraging employees to make decisions, and sharing information. Future research should investigate more concrete interventions to promote “judgment” and “kindness” in female employees, and “teamwork” and “creativity” in male employees in the workplace.

### Limitations

This study has several limitations. First, although previous studies have discussed the importance of character strengths, few have examined the biological basis and scientific meaning of these strengths [6, 46–48] . Second, the present study did not collect information on certain sociodemographic factors (e.g., marital status and childcare burden), psychiatric comorbidities (e.g., substance use, anxiety, insomnia, and other such disorders), and other work-related factors (e.g., work hours per week, duration of current job, duration at current workplace, work environment, and other examples) that potentially affect depression. In addition, major risk factors for suicidality (e.g., family history of suicide, childhood maltreatment, personality disorder, and other factors) were also not examined in the present study. Third, this was a cross-sectional study. Although strength is trait-like and is considered as a causal factor, there may be an error in causality (e.g., effect of years of education on intellectual strengths). Fourth, there are limitations in generalizing and applying these research results to various occupations and races. The present participants were from a homogeneous occupational and social environment, because they were employees of two public companies located in Seoul. Fifth, because we collected the data using a self-report questionnaire, reporting bias should be considered in interpreting the results. In addition, we used the K-MINI for evaluating suicidality in this study, because this tool has been validated in Korea [16] and it has been used in a national suicide prevention project. However, a recent systematic review and meta-analysis reported that the positive predictive value of suicidal behaviours using clinical instruments was inadequate, and no “high-risk” classification was clinically useful [49]. Finally, there could be nonresponse bias despite our effort to avoid it, such as sufficient data collection periods (one month), ensuring confidentiality, and sending reminders to potential respondents. Therefore, to ensure the generalizability of our results, further research with a longitudinal design, a larger number of participants, objective assessment methods, and control for potentially confounding variables is necessary.

## Conclusion

Based on the results of the present study, we suggest that focusing on “judgement” and “kindness” in female employees, and “teamwork” and “creativity” in male employees, as well as engaging in activities that use these strengths at the workplace can be protective against depression and suicidality. Future research should focus on developing interventions to promote these character strengths among employees in the workplace.

## Abbreviations

BDI-II: Beck Depression Inventory
K-MINI: Korean version of the MINI International Neuropsychiatric Interview (MINI) suicidality module
LFPR: Labour Force Participation Rate
OECD: Organization for Economic Co-operation and Development
TCI: Temperament and Character Inventory
VIA-72: 24 Character Strength Alphas of the Values in Action (VIA) Survey-72
